# The Relationship of Food to Scorbutis in Children

**Published:** 1893-04

**Authors:** E. F. Brush

**Affiliations:** Mt. Vernon, N. Y.


### The Relationship of Food to Scorbutus in Children.

BY E. F. BRUSH, M.D., OF MT. VERNON, N. Y.

Read in the Section of Diseases of Children, at the Forty-third Annual Meeting of the American Medical Association, held at Detroit, Mich., June, 1892.

The question of scurvy in infancy is, I think, one of the phases of children's diseases that has been lost sight of very largely in the study of pediatrics. There is no doubt but that the disease exists in infancy to a greater extent than we are aware, or should infer from medical literature. There can be little doubt when your attention is called to the fact, but that you will agree that many of the cases reported as rickets and marasmus, should be classified as scorbutus. Dr. Northrup, of New York, read a paper on scorbutus last September, in Washington, before the Pediatrics Society, and I think much good will come from this paper, which was a very interesting and instructive one, calling as it does the attention of pediatricians to the fact that such a disease occurs in infancy, and that it is characterized by very nearly the same pathological phenomena as is the disease in adult life. Now as this affection in the adult is well recognized, and the cause pretty well understood, I think it would not be amiss for me to call attention to some of the conditions in adult life that stand in a causative relation to the development of scurvy, and then we can see how prone to the affection the artificially fed infant of to-day must be. Every living creature on earth, or in the water thereof, subsists on matter that was once alive, and all food with the exception of a few condiments has been living, growing matter at one time. I think it can be safely affirmed that man is the only animal who not only kills his food, but makes it absolutely dead and sterile before he consumes it for his nourishment. The old adage, "that God sends the food, but the devil sends the cook," means more than has been credited to the saying. It has always been supposed that the unpalatable preparation of food was the devilish cooks' work, but

I believe the cook whom the devil has sent to mislead us is the chemist. With his retort and his balances, with his reagents and his surmises, he has led us to believe that God has failed to do his work properly by uniting the nutritive materials in a living form, and possessed with a vitality that the chemist knows nothing about. He has taken up a deal too much room, and made things altogether too complicated. The chemists would have us believe that man needs for his nourishment only so much nitrogenized and so much non-nitrogenized matter; that the material with which the Creator has united these, and the vitality with which he has endowed it, is totally unnecessary. According to the advanced ideas of the chemist, a little jar of Liebig's extract of beef is far better than ten pounds of meat in the form we find it in nature. Milk that has been skimmed, dried and powdered, and mixed with a little cocoa butter, is far better than fresh milk from the laboratory of nature as the Great Giver has allowed us to procure it. I remember seeing it stated in some standard work, that when the chemist had reached his goal, armies would be able to carry in a small vial all the nourishment that was needed for their support for several days, instead of the weighty load of bread and meat which they are now obliged to carry.

I tell you gentlemen, the chemist has tried, to be too smart, and we have been duped by him in too many instances. Every living creature requires for his proper nourishment some raw living food. Every young living creature needs living food. The mammalia all take it direct from the living fountain, and young feathered tribes are supplied by their parents with living creatures for food. Even the young fishes consume the living animalculæ, and man seems to be the only one of God's creatures who thinks he knows better. The artificially fed infant gets his food from the knowing chemist, or must according to the prevailing fashion, have all the life sterilized out of it, if he gets any as nature supplies it without the intervention of the chemist. I am positive there is more in organism, and the vitality that holds it together, than is dreamed of in our advanced chemical knowledge.

We know that the sea-faring man, the soldier, the peasant of a famine stricken district, are all subject to scurvy when they are deprived of fresh living food, and any growing vegetable, animal matter or other material not dead, will cure the disease. Milk retains its anti-scorbutic qualities for a few hours, so does meat after the animal is subjected to what we call killing. I suppose the muscular response to electric excitability will indicate the point at which meat retains its vitality after the animal has been killed, and I think it is a well known fact that vitality is the anti-scorbutic quality of a food. Now if the absence of this quality for any given time from the nutritive material of an individual, produces such dire results as scurvy, it must follow that this principle is a necessary condition for proper nourishment, and the chemist's idea of proximate principles being the only necessity for nutriment, must be a fallacy. Of course neither the medical man nor the chemist knows what vitality is, but it is with us everywhere, and by reason of its possession, we ourselves live and move, and have our being, and every article of food we consume was gathered together, and formed into nutritive food for us by the living and growing quality of vitality. Is it unreasonable to suppose then, we can get from this quality some sort of force that is necessary for our well being, and are we not depriving ourselves of some absolute necessity, when we eliminate every vestige of vitality from our daily bread? Since I have been possessed with this idea, I have observed several cases of simple dyspepsia recover completely by the use of live raw food. A few months ago, a patient came to me complaining of violent attacks of vertigo and temporary blindness. He had been losing flesh and strength for several months. I examined his urine, and found it to contain about 15 per cent. of albumen. He was directed to take nothing but living raw food—that is, milk not over four hours old, eggs laid but a few hours, raw oysters, raw clams, lettuce and other greens with no dressing, meat within a few hours after it was killed, eaten raw, apples, oranges and other raw fruits. No medicine except three drops of the tincture of nux vomica in water before meals as a placebo. At the end of fifty days the albumen had entirely disappeared. He had but few attacks of vertigo after he had the exclusive diet, and the attacks of temporary blindness never returned, and now after five months, he is in a better state of physical health than he had been before for years.

Now this one case proves very little, but it at least indicates that the diet must have had some influence in the man's recovery. I do not affirm that albuminuria always arises from the same cause, neither do I think any will affirm that defective nutrition is not one of the prominent etioligical factors in Bright's disease, and if I am right in my deductions, one of the defects in our materia alimenteria is the absence of vitality; and if this is true regarding the adult, how much more must it be so with the luckless infant deprived of the normal living fountain which nature designed for its proper nutrition. I am firmly convinced that not only scorbutus and other serious affections arise from the absence of living food in the infant, but many of the weakly non-resisting babes succumb to disease, or live with more or less suffering, because of the absence of vitalized food.

One of the defects of the finite mind is its limit to be able to only harbor one idea at a time, and so when one man discovers a truth he straightway imagines that he has solved all the mysteries of the science relating to the subject to which the discovered truth relates. I am willing to acknowledge that the vitality of a food is not its only requirement, but I am thoroughly convinced that it is one of the very important necessities to perfect nutrition. There can be little doubt that many children live who have been so improperly nourished (although fat) that life is more or less of a burden. A great deal of this may be due to their progenitors, their surroundings, and many other conditions, but some suffer by reason of improper food, and the method of its administration.

The French nation have as an executive officer in their scheme of government, a medical Health Officer. He has lately discovered that the death-rate in infants is so alarmingly large, that there is great danger of depeopling the nation, therefore he has issued an edict forbidding the use of the long tube nursing bottle in feeding infants, and I suppose he imagines that by this wise (?) law he will repeople France. If the death-rate could only be lowered by so simple an edict, our occupation would be gone; but we know that there are many other conditions, graver, more important and serious, than simply the form of a nursing bottle. There are very many things for us to find out before we know it all, but one fact I feel sure of, that the constant use of dead food with an infant is wrong. There is no greater field for the pediatrician, than the study of infant feeding. We must exclude from our councils, absolutely, the patent baby food manufacturer, and study how we can get a full supply of fresh, raw, living food to the unlucky infant who has to submit to an artificial diet, and then from the knowledge we are now in possession of, we will know that we are guarding the young, at least, from the danger of scurvy, and perhaps greater evils.

